# WhatsApp as a tool for psychiatric crisis management: lessons from a Ghanaian support group

**DOI:** 10.1186/s12888-025-07068-9

**Published:** 2025-07-11

**Authors:** Gideon Prempeh Owusu, Semefa Acheamponmaa Yawa Dordoye, Seyram Ametorxe Maloe Abra Asigbey, Eugene Kobla Dordoye

**Affiliations:** 1 Department of Psychological Medicine, Ho Teaching Hospital, Ho, Ghana; 2 Thunderbird School of Global Management, Arizona State University, Downtown Phoenix, AZ USA; 3 School of Medicine, University of Health and Allied Sciences, Ho, Ghana; 4 Department of Psychiatry, Zucker Hillside Hospital/Northwell Health, Glen Oaks, NY USA

**Keywords:** WhatsApp, Psychiatric emergencies, Digital mental health, Peer support, Ghana, Crisis intervention

## Abstract

**Background:**

Psychiatric emergencies, such as psychosis, suicidality, and severe agitation, pose significant challenges in low-resource settings like Ghana, where access to timely care is often limited. Digital communication platforms, particularly WhatsApp, offer promising avenues for supplementing psychiatric support.

**Methods:**

This qualitative program evaluation examined a WhatsApp-based mental health support group operating in Ghana over a ten-year period. The group included mental health service users (MHSUs), caregivers, and professionals. Data were derived from archived chat logs, facilitator notes, and participant reflections. Thematic analysis of data was guided by digital ethnography and narrative reconstruction.

**Results:**

Over the course of a decade, the group successfully averted more than 20 documented psychiatric crises with only one hospitalization reported. The platform enabled early symptom recognition, peer and professional intervention, and timely referrals. The ten MHSUs who contributed reflections were predominantly female, with bipolar disorder being the most common diagnosis.

**Conclusion:**

WhatsApp-based support groups can serve as vital adjuncts to formal psychiatric care, offering a scalable, community-driven model for crisis monitoring and intervention. The success in Ghana demonstrates the value of integrating digital platforms into national mental health strategies, supported by ethical guidelines and professional oversight. Future studies should evaluate long-term outcomes and explore replication in similar low-resource contexts.

**Clinical trial number:**

Not applicable.

## Background

Psychiatric emergencies, characterized by acute disruptions in behavior, mood, cognition, or perception, pose significant risks to individuals and their communities. Conditions such as suicidality, severe agitation, and acute psychotic episodes demand immediate attention to prevent harm and promote stabilization [1]. Individuals living with mental illness, as well as their caregivers, derive significant benefit from supplementary follow-up beyond standard clinic appointments. However, in resource-limited settings such as Ghana and other low- and middle-income countries (LMICs), the feasibility of such ongoing support is often constrained by multiple systemic barriers. These include the financial burden of care, persistent stigma surrounding mental illness, critical shortages of mental health professionals, and inconsistent availability of family and community-based support [2]. Addressing these challenges is essential for sustaining long-term recovery and reducing relapse risk in these vulnerable populations.

Within this context of constrained formal mental health services, digital technologies offer promising avenues for augmenting psychiatric care [3]. *WhatsApp* stands out in the Ghanaian context among digital communication platforms due to its widespread adoption, accessibility, and capacity for real-time interaction. Compared to other social media applications, WhatsApp offers end-to-end encryption, sustained connectivity, and flexible communication formats (including text, voice, and video), rendering it particularly well-suited for mental health engagement in settings where traditional service delivery remains constrained [4].

Emerging literature suggests that digital peer support, moderated by professionals, can serve as a viable supplement to in-person care [4]. Studies from diverse regions have reported improved mental health literacy, reduced isolation, and enhanced coping among users of mental health support groups on digital platforms [5, 6]. Yet, empirical evidence from LMICs, particularly in Africa, specifically for mental health, remains sparse. This gap is particularly relevant given the rapid adoption of mobile technologies in sub-Saharan Africa and the persistent treatment gap in mental health services [7].

This study addresses a critical gap in the literature by evaluating a decade-long experience with a WhatsApp-based mental health support group in Ghana. Uniquely developed through the collaborative efforts of service users and mental health professionals, the initiative represents a grassroots model of community-based crisis monitoring, peer support, and psychiatric intervention delivered through a digital platform. The primary objective of this program evaluation is to document WhatsApp’s utilization for psychiatric crisis management, characterize the types of interventions implemented, and distill key lessons from this innovative approach. The study contributes valuable insights to the growing discourse on integrating digital technologies into mental health service delivery in low- and middle-income countries (LMICs).

## Methods

### Study aim, design, and setting

This study explores the role of WhatsApp as a platform for managing psychiatric crises in a Ghanaian mental health support group. It evaluates its effectiveness in crisis identification, early intervention, and continuous psychiatric support within an informal mental health network. Employing a qualitative case-based program evaluation, the study was situated within a longitudinal observational framework. It drew on digital ethnography principles and retrospective reconstruction of interactions and events spanning a ten-year period (2013–2023).

### Participants and study context

The Mental Health Support Group was initially established by two individuals: one diagnosed with bipolar disorder and the other a spouse of a patient with schizophrenia, along with their treating psychiatrist. Over the ten-year period, approximately 30 mental health service users (MHSUs) have participated in the WhatsApp support group. As of the time of evaluation, the group had expanded to one hundred and sixteen (116) members, including MHSUs, caregivers, and mental health professionals. For the purposes of this evaluation, 10 MHSUs who had participated in the group and were willing to provide reflections were included. These individuals consented to contribute to the study through retrospective narrative accounts and thematic reflections.

### Interventions and procedures

The support group initially held monthly in-person meetings at a member’s home before relocating to Legon Hall Chapel at the University of Ghana. These meetings centered on sharing lived experiences related to specific psychiatric conditions and exchanging coping strategies. Structured educational sessions were also organized to enhance participants’ understanding of mental health disorders and available support systems. As logistical and accessibility challenges increased, the group transitioned to WhatsApp as its primary platform.

**Fig. 1:**
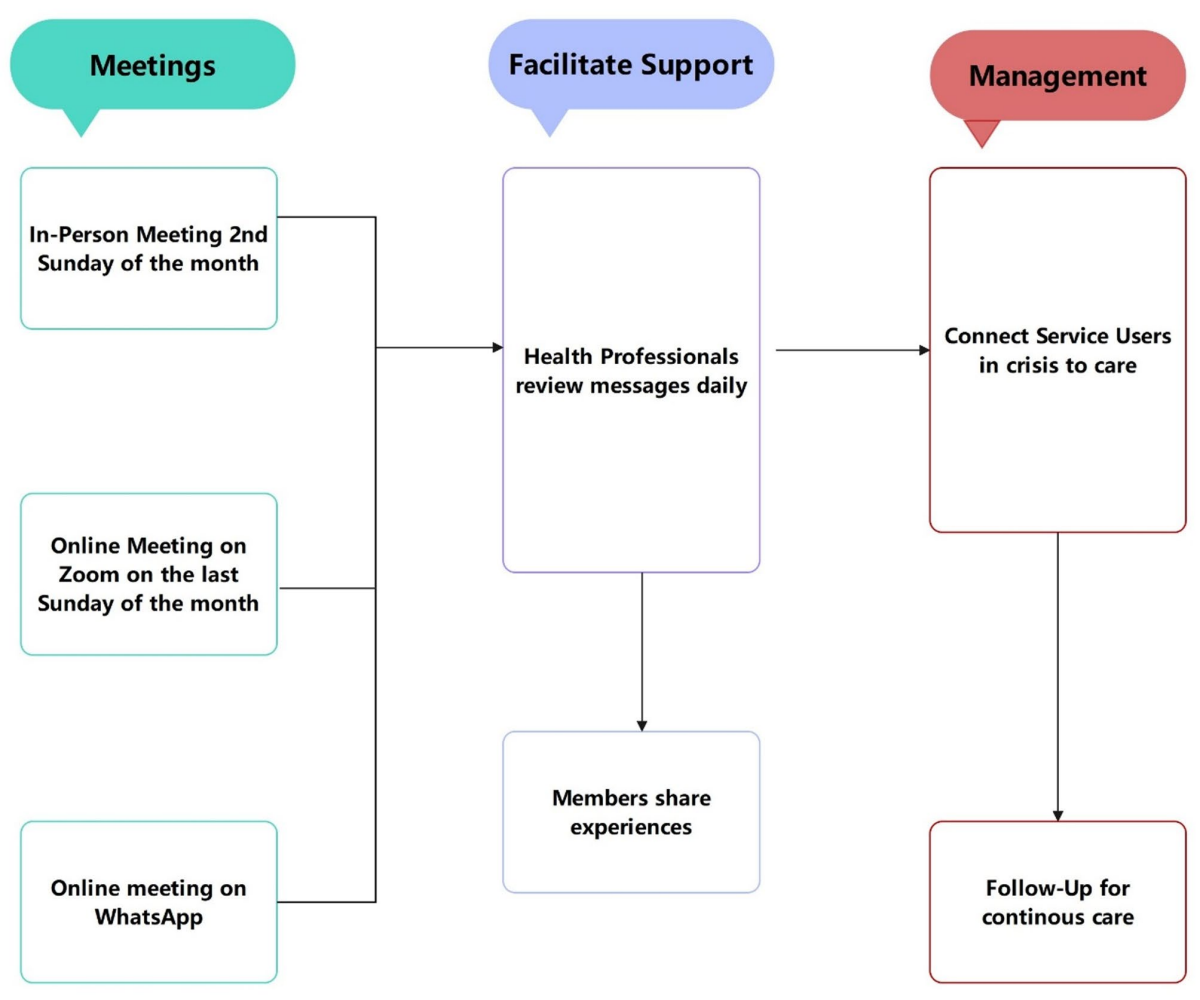
Flowchart 1: Operational framework of the whatsapp mental health support group

This digital shift enabled real-time emotional support, psychiatric referrals, and crisis intervention. Information typically flowed from the recognition of a crisis (often identified through behavioral changes or distress signals in WhatsApp messages), to initial peer or professional detection, and subsequently to referrals or direct interventions. Crisis management operated without external funding, relying solely on volunteerism and the goodwill of mental health professionals (see Flowchart, Fig. 1).

### Data analysis

Data sources included archived WhatsApp chat logs, facilitator records, case reflections, and participant narratives. These were analyzed retrospectively, with all identifiable information anonymized. Manual coding techniques were applied to identify emergent themes such as symptom escalation, supportive communication, and intervention pathways. Though formal discourse analysis was not employed, the analysis drew from narrative elements and group interaction patterns to elucidate how crises were managed.

### Ethical considerations

All participants provided informed digital consent for participation and data use. The group operated with strict confidentiality norms. Ethical concerns around mixed patient-provider communication, data privacy, and consent were mitigated through ongoing reminders, anonymization protocols, and voluntary disclosure practices. No formal institutional review board approval was sought due to the retrospective and evaluative nature of the study, but ethical standards consistent with digital health research were upheld.

## Results

Over the ten-year period of its operation, the WhatsApp-based support group effectively averted more than twenty (20) documented psychiatric crises that, without timely intervention, may have escalated into emergencies necessitating hospitalization or posing a significant risk of harm. These crises included acute psychotic episodes, suicidal ideation, homicidal behavior, manic episodes, and severe anxiety attacks. Notably, only one instance required inpatient psychiatric admission, highlighting the preventive potential of this digital peer-support intervention model.

The ten MHSUs who contributed reflections to this evaluation were predominantly female (60%), with most participants aged between 32 and 35 years. Half of the respondents had a primary diagnosis of bipolar disorder, while others were living with schizophrenia (30%) or depression (20%). Most had experienced their condition for less than ten years, and the majority were either employed (40%) or self-employed (50%). Half of the participants resided in Accra, with others located in Tema, Ho, and Cape Coast. A demographic table (Table 1) presents details for mental health service users such as gender, diagnosis, employment status, length of illness, and geographic distribution.

**Table 1:** Socio-demographic characteristics of participating mental health service users (*N* = 10)

Variable	Category	Frequency	Percentage (%)
Age	< 32	3	30.0
	32–35	4	40.0
	> 35	3	30.0
Gender	Male	4	40.0
	Female	6	60.0
Diagnosis	Bipolar Disorder	5	50.0
	Schizophrenia	3	30.0
	Depression	2	20.0
Duration of Illness	< 5 years	4	40.0
	5–10 years	4	40.0
	> 10 years	2	20.0
Employment Status	Employed	4	40.0
	Self-employed	5	50.0
	Unemployed	1	10.0
Current Residence	Accra	5	50.0
	Tema	2	20.0
	Ho	2	20.0
	Cape Coast	1	10.0

Participant narratives offer direct insight into the perceived value of the group. One member, a mental health service user (MHSU), recounted:

*“When I started getting thoughts that I could fly and wanted to try jumping from my roof*,* I told the group. Within minutes*,* I was on a video call with a doctor*,* and my brother was called to check on me.” [MHSU*,* Male*,* 26]*.

This early identification of a manic episode enabled rapid intervention, preventing potential injury. Another caregiver shared,

*“There was a day my son started speaking to voices again. I posted it on the platform*,* and within an hour we had adjusted his medication with the doctor’s advice. He calmed down that same evening.” [Caregiver*,* Female*,* 47]*.

This rapid response mechanism prevented a full psychotic relapse. From a facilitator’s perspective, one psychiatrist reflected,

*“We’ve seen people post subtle signs*,* withdrawal*,* changes in tone*,* and with gentle probing*,* they open up. It’s amazing how many crises have been caught this way before they explode.” [Psychiatrist*,* Male*,* 55]*.

Routine weekly check-ins facilitated ongoing surveillance, while ad-hoc messages flagged urgent situations. The peer structure allowed members to monitor each other informally, identifying deviations from baseline behavior.

A particularly notable case involved a young adult who, during the COVID-19 pandemic, expressed feelings of entrapment and suicidal ideation. Immediate peer responses, followed by psychiatric intervention arranged through the group, prevented a suicide attempt. As the individual later shared,

*“I didn’t think anyone would care. But I got more support from this group than I expected. I’m alive because someone listened.” [MHSU*,* Female*,* 32]*.

In one illustrative case, a 27-year-old MHSU with bipolar disorder began expressing paranoid delusions on the platform, alleging that international security agencies, such as the CIA, were targeting him. A Ghanaian psychiatrist based in Maryland, USA, recognized this as an acute manic episode and coordinated with a local psychiatrist in Ghana. Through these efforts, the individual was promptly referred and admitted to a psychiatric facility for treatment.

In another incident, a 28-year-old woman with schizophrenia posted disorganized and threatening content that concerned the group. A psychiatrist member identified the episode as a resurgence of psychosis with homicidal ideation. The group mobilized quickly to contact family members and facilitate medical intervention. The timely response prevented a potential act of violence and enabled her continued employment post-crisis.

The structure of the group allowed for decentralized monitoring, immediate feedback, and access to professional consultation without the logistical and financial barriers typical of traditional care settings. The collaborative efforts between users and professionals underpinned the high rate of successful de-escalation of crises.

While precise metrics were limited by the informal nature of data capture, qualitative accounts and facilitator records consistently emphasized timely intervention and symptom containment. The group’s singular hospitalization instance over a decade strongly suggests the utility of WhatsApp as a tool for early detection and coordinated psychiatric response.

## Discussion

This study demonstrates that WhatsApp can serve not merely as a messaging platform, but as a structured tool for psychiatric surveillance and crisis response. Operating for over a decade, the support group successfully averted numerous psychiatric crises through the collaborative vigilance of mental health service users (MHSUs), caregivers, and professionals. These findings are particularly significant in the Ghanaian context, where traditional modes of crisis management, such as hotlines and in-person interventions, have limitations regarding accessibility, reach, and scalability. On the other hand, WhatsApp offers an opportunity to engage individuals in distress, connect them with mental health professionals, and provide access to immediate support networks [8, 9].

WhatsApp, considered a social media application, enables the creation and sharing of user-generated content while facilitating virtual connections between individuals. Whereas the negative impact of social media on mental health raises significant concern across the globe, this tool has also been powerfully adopted to facilitate healthcare in general and mental health in particular in some parts of the world [10, 11]. Facilitation of social integration, retention in mental health services, and peer support networks have been noted as broad benefits of social media in mental health delivery [8]. Onyeaka et al. showed apparent beneficial effects of social media use among people with depression and anxiety in a large cross-sectional survey [12].

The blended composition of the group allowed for rapid detection of early warning signs and timely intervention. Weekly check-ins and real-time discussions cultivated a form of community-based surveillance in which peers flagged concerns and professionals responded swiftly. This structure enabled a level of responsiveness rarely achievable in conventional clinical settings, particularly in under-resourced environments.

What distinguishes this initiative from other digital mental health efforts is its long-term continuity, peer-professional integration, and consistent, documented prevention of psychiatric escalation. Existing literature supports the utility of WhatsApp in improving mental health literacy and peer support, particularly among caregivers [8]. However, there is limited documentation of its sustained use in active psychiatric crisis management involving clinician engagement. In this regard, the current study adds novel evidence to the field.

The model’s success also demonstrates the capacity for diaspora professionals to contribute meaningfully to mental health care in their countries of origin. WhatsApp’s real-time functionality and broad accessibility enabled geographically dispersed clinicians to play a pivotal role in guiding local interventions and effectively bridging care gaps.

These findings align with broader literature on digital mental health tools in low- and middle-income countries (LMICs), which suggest such platforms are cost-effective, scalable, and acceptable [13]. However, the proactive and longitudinal nature of this group sets it apart from many other interventions that tend to be episodic or passive.

Despite its strengths, this study has several limitations. As a qualitative program evaluation, the findings are based on retrospective narrative accounts and thematic analysis of chat logs, without structured interviews or quantitative outcome measures. This constrains the generalizability of the results and precludes statistical evaluation of the intervention’s impact. Also, the informal nature of the platform, while facilitating real-time communication, did not support systematic data capture, leading to the likely underreporting of crises or variations in participant experiences.

Participant engagement in the evaluation process was another constraint, as only a subset of mental health service users consented to share reflections. Technological barriers, including intermittent internet access, limited smartphone capabilities, and data affordability, sometimes hindered active participation or delayed responses during critical moments. The group’s sustained reliance on volunteer mental health professionals, in the absence of external funding or institutional support, occasionally resulted in facilitator fatigue and lapses in moderator availability.

Another limitation lies in the absence of a control group or pre-/post-intervention design, which limits causal inferences about the effectiveness of the WhatsApp support group. Moreover, participant anonymity and pseudonymization, while ethically necessary, impeded detailed demographic tracking or longitudinal outcome monitoring. Ethical review was not formally obtained due to the retrospective and anonymized nature of the data, though ethical standards consistent with digital health research were maintained.

## Conclusion

Integrating WhatsApp-based support groups into the formal psychiatric care system has the potential to significantly strengthen mental health service delivery. Such platforms facilitate ongoing engagement, patient education, and timely crisis intervention, ultimately improving access to care and reducing the incidence of psychiatric emergencies.

The Mental Health Authority could play a key role in organizing or endorsing WhatsApp-based initiatives, ensuring that community mental health practitioners across all districts actively participate. This would facilitate ongoing patient monitoring, timely interventions, and enhance community engagement in mental health awareness.

To safeguard patient confidentiality, it is essential to implement strict ethical guidelines governing the use of these platforms. Educating both practitioners and community members on privacy regulations, ethical responsibilities, and restrictions on the public sharing of sensitive information would be critical to maintaining a secure and professional online support environment.

Future studies should explore replicability and integration into national mental health strategies. Further research should assess the long-term outcomes of social media-driven psychiatric interventions.

## Data Availability

The datasets generated and/or analyzed during the current study are not publicly available due to confidentiality considerations, but are available from the corresponding author on reasonable request.
